# Hydrological asymmetry and water stress in Peru: An integrated assessment of resource distribution, anthropogenic pressure, and governance gaps across three drainage basins

**DOI:** 10.12688/f1000research.176208.1

**Published:** 2026-03-30

**Authors:** Juan Eduardo Suarez Rivadeneira, Freddy A Manayay, Jhon Danilson Campos Mego, Wilfredo Ruiz Camacho, Italo Maldonado Ramirez, Gustavo Adolfo Perez Londoño

**Affiliations:** 1 Mechanical and Electrical Engineering Professional School, National University Toribio Rodríguez de Mendoza of Amazonas, Bagua, 0172, Peru; 2 Biosystems Engineering Professional School, National University Toribio Rodríguez de Mendoza of Amazonas, Chachapoyas, Amazonas, 01001, Peru

**Keywords:** Integrated Water Resources Management; Hydrological Cycle; Water Stress; Groundwater Recharge; Water Governance; Economic Compensation; Peru.

## Abstract

**Background:**

Peru presents a paradigmatic case of hydrological asymmetry, where national water abundance (56,887 m ^3^/capita/year) masks acute regional scarcity. The Pacific basin, hosting 66.4% of the population, possesses only 1.9% of renewable water resources (1,628 m ^3^/capita/year), falling below the Falkenmark water stress threshold (1,700 m ^3^/capita/year). Conversely, the Amazon basin holds 97.8% of resources for 30.4% of the population (183,142 m ^3^/capita/year). This 112.5-fold asymmetry ratio exceeds comparable international cases (Egypt: 49.2; China: 8.0), yet Peru lacks systemic redistribution mechanisms.

**Policy and Implications:**

Current governance frameworks under Law 29338 (2009) have failed to address territorial misalignment between supply, demand, and institutional capacity. Economic compensation mechanisms (S/205.5 million annually) paradoxically tax scarcity rather than value ecosystem services, with the Pacific basin contributing 61.7% of revenues despite extreme resource constraints. Groundwater extraction in coastal regions operates at 100% of renewable recharge limits, indicating unsustainable fossil aquifer mining. Climate projections indicate 30% runoff reduction in glacier-fed Pacific basins by 2100, exacerbating existing stress.

**Recommendations:**

(1) Implement basin-scale demand management and irrigation efficiency programmes in the Pacific basin; (2) Strengthen groundwater monitoring and abstraction controls to prevent irreversible depletion; (3) Reform economic compensation mechanisms to incorporate ecosystem service valuation, redistributing revenues toward hydrologically strategic headwater regions; (4) Integrate indigenous water governance systems (comunidades campesinas, ayllus) into state planning frameworks; (5) Establish inter-basin transfer feasibility studies given the extreme asymmetry ratio.

**Conclusions:**

Peru’s water challenge stems from governance asymmetry layered upon hydrological asymmetry, not absolute scarcity. Without institutional mechanisms reconciling hydrology, demography, and political economy, climatic variability will entrench coastal scarcity while Amazonian abundance remains underutilised. The integrated assessment framework presented provides transferable insights for water-rich yet spatially asymmetric nations facing increasing climatic variability.

## 1. Introduction

Water scarcity affects 4 billion people globally, with physical scarcity (insufficient resources) and economic scarcity (inadequate infrastructure) representing distinct governance challenges. ^1, 2^ The Falkenmark Water Stress Index remains the standard metric for physical scarcity assessment, though composite indices (Water Poverty Index, Global Water Security Index) provide multidimensional perspectives. ^3– 6^ Nations facing extreme hydrological asymmetry—Egypt (Nile dependence, 55 m ^3^/capita/year renewable resources), Israel (arid climate, 230 m ^3^/capita/year), and China (North-South disparity, 8-fold difference)—have implemented diverse governance responses ^7– 9^: inter-basin transfers, desalination, and virtual water trade. ^7, 10^

Peru’s hydrological configuration presents a unique case: national abundance (56,887 m ^3^/capita/year) coexists with regional scarcity (1,628 m ^3^/capita/year in Pacific basin). ^11, 12^ This “scarcity amidst abundance” paradox distinguishes Peru from uniformly water-stressed nations and demands tailored governance approaches. ^12, 13^

Peru’s territory (1,285,216 km ^2^) encompasses three major drainage basins: Pacific (278,482 km ^2^, 21.7%), Amazon (957,823 km ^2^, 74.5%), and Titicaca (48,911 km ^2^, 3.8%). ^14, 15^ The 2025 population (34,038,457) is distributed highly unevenly: 66.4% in the Pacific basin, 30.4% in Amazon, 3.2% in Titicaca. ^16^ This demographic pattern inverts hydrological endowment: the Pacific basin possesses 1.9% of water resources; the Amazon basin holds 97.8%. ^17, 18^

The Pacific basin’s per capita availability (1,628 m ^3^/capita/year) falls below the water stress threshold (1,700 m ^3^/capita/year), positioning it alongside traditionally water-scarce regions. ^19, 20^ However, the Amazon basin’s abundance (183,142 m ^3^/capita/year) creates national average availability (56,887 m ^3^/capita/year) that masks critical regional stress. ^19, 21^

Integrated Water Resources Management (IWRM), formalised at the 1992 Dublin and Rio conferences, emphasises cross-sectoral coordination and multi-level governance. ^22, 23^ Peru’s Law No. 29338 (2009) established the National Water Authority (ANA) as the IWRM implementing institution, with subsequent regulations addressing hydraulic infrastructure, economic compensation, and watershed councils. ^17, 22^

Despite institutional development, implementation gaps persist. Groundwater remains inadequately characterised (only 31% of 159 aquifers studied). ^14, 18^ Economic compensation mechanisms collect S/. 205.5 million annually but distribute revenues inequitably. ^24, 25^ Climate change projections indicate 30% runoff reduction in the Pacific basin by 2100 due to glacier retreat. ^26^

This study addresses four research questions: 1. What is the magnitude and uncertainty of hydrological disparities across Peru’s basins? 2. How do these disparities compare internationally? 3. What governance mechanisms have failed to address the asymmetry? And 4. What policy interventions can achieve hydrological equity?

## 2. Materials and methods

### 2.1 Study area and data sources

*Pacific Basin*: Arid coastal region (precipitation: 0–200 mm/year in lowlands, 1,000–2,000 mm in Andean headwaters). Population: 22,601,357 (2025). Water resources: 36,804 million hm ^3^/year.

*Amazon Basin:* Tropical rainforest (precipitation: 2,000–3,000 mm/year). Population: 10,336,549. Water resources: 1,893,055 million hm ^3^/year.

*Titicaca Basin:* High-altitude plateau (3,600–4,500 m elevation). Population: 1,100,551. Water resources: 6,468 million hm ^3^/year.

Data sources: ANA hydrological records (2024), INEI census (2017) and projections (2025), SNIRH monitoring network.

### 2.2 Analytical methods

**Falkenmark Water Stress Index (WSI):**

$$\mathrm{WS}I_{i}=\frac{\mathrm{TRW}R_{i}}{P_{i}}X10^{6}$$

Where $\mathrm{WS}I_{i}$ = Water Stress Index for basin i (m ^3^/capita/year), $\mathrm{TRW}R_{i}$ = Total Renewable Water Resources (hm ^3^/year), $P_{i}$ = Population.

**Uncertainty Quantification:** Monte Carlo simulation (n = 10,000 iterations) with parameter distributions:

• Population: Normal distribution, σ = 2% of mean (census uncertainty)
• Water resources: Triangular distribution, ±15% range (measurement error)
• Extraction rates: Uniform distribution, ±10% range

Degree of Pressure (DP):

$$DP_{i}=\frac{\sum_{s}U_{i,s}}{\mathrm{TRW}R_{i}}X100$$

Where $U_{i,s}$ Water use in sector s (agricultural, industrial, population, energy) in basin i .

**Mann-Kendall Trend Test:** Applied to time series data (1990–2025) to detect monotonic trends in water stress indicators ^27^:

$$S=\sum_{i=1}^{n-1}\sum_{j=i+1}^{n}\mathrm{sgn}(x_{j}-x_{i})$$

Where sgn() is the sign function, x = annual WSI values.

**Comparative Analysis:** International comparison using standardised metrics:

• Asymmetry Ratio: $\mathrm{AR}=\frac{\mathrm{WS}I_{\max}}{\mathrm{WS}I_{\min}}$ across subnational units
• Water Poverty Index (WPI) components ^28, 29^

### 2.3 Political ecology framework

Governance failure analysis employed the “hydrosocial cycle” concept, examining how political and economic power relations shape water distribution. ^30^ Key dimensions:

• *Accumulation by dispossession:* Historical concentration of water rights in export agriculture and mining
• *Scalar politics:* Mismatch between watershed boundaries and administrative jurisdictions
• *Knowledge politics:* Exclusion of indigenous water management systems from state planning

## 3. Results

### 3.1 Hydrological disparities with uncertainty quantification

Monte Carlo simulations reveal significant uncertainty ranges ( Table 1). The Pacific basin WSI (1,628 m ^3^/capita/year) has 95% CI of 1,512-1,744, confirming water stress conditions even under optimistic scenarios. The Amazon basin WSI (183,142 m ^3^/capita/year, 95% CI: 155,171-211,113) maintains abundance across all simulations.

**Table 1.** Water stress indicators with uncertainty ranges (2025).

Basin	WSI mean	95% CI lower	95% CI upper	DP mean (%)	95% CI DP	Falkenmark status
Pacific	1,628	1,512	1,744	75.8	72.3–79.4	Scarcity
Amazon	183,142	155,171	211,113	0.5	0.4–0.6	No stress
Titicaca	5,877	4,995	6,759	7.0	6.3–7.7	No stress

**Figure 1.**
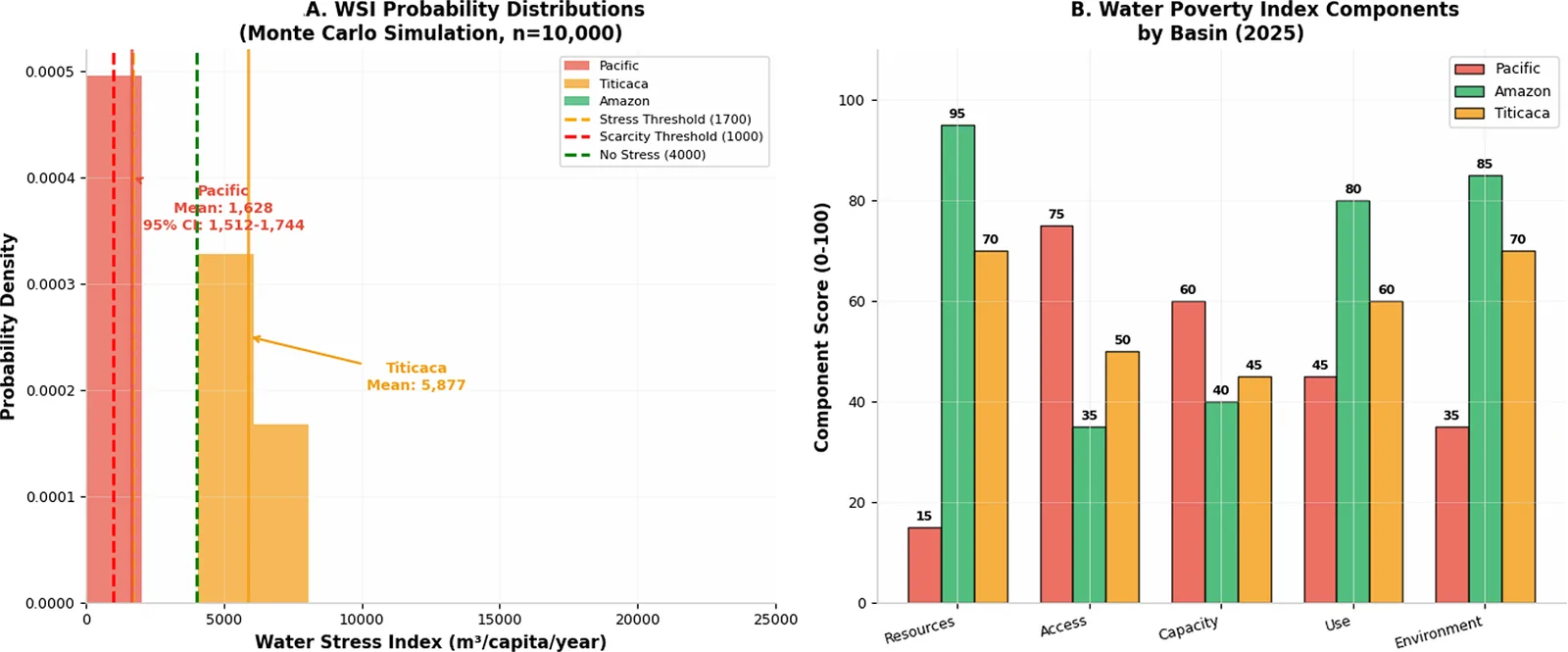
Water stress index probability distributions with uncertainty quantification. (A) Monte Carlo simulation (n = 10,000) showing WSI probability distributions for Pacific (red), Amazon (green), and Titicaca (orange) basins with 95% confidence intervals. Dashed vertical lines indicate Falkenmark thresholds: scarcity (1,000 m ^3^/capita/year), stress (1,700 m ^3^/capita/year), and no stress (4,000 m ^3^/capita/year). Pacific basin mean WSI: 1,628 (95% CI: 1,512–1,744). Amazon basin mean WSI: 183,142 (95% CI: 155,171–211,113). (B) Water Poverty Index components by basin (0–100 scale, higher = better). Pacific basin shows low Resources scores (15) but high Access (75); Amazon basin shows inverse pattern with high Resources (95) but lower Access (35).

The WSI distribution of the Pacific basin ( Figure 1A) shows a mean of 1,628 with a 95% confidence interval between 1,512 and 1,744. This indicates that the majority of simulations (>95%) exceed the 1,000 scarcity threshold, and there is uncertainty regarding whether the stress condition crosses the critical 1,700 threshold, as the upper portion of the distribution reaches values higher than this limit.

### 3.2 International comparative analysis

Peru’s asymmetry ratio (AR = 112.5) exceeds comparable cases ( Table 2):

**Table 2.** International comparison of hydrological asymmetry.

Country	Basins/Regions	WSI range (m ^3^/capita/year)	Asymmetry ratio	Primary response
Peru	Pacific/Amazon	1,628/183,142	112.5	None implemented
Egypt	Nile/Desert	650/32,000 *	49.2	Virtual water import
China	North/South	500/4,000	8.0	South-North Water Transfer
Spain	Ebro/Segura	2,500/15,000	6.0	Inter-basin transfers
USA	Colorado/Mississippi	1,200/8,500	7.1	Colorado River Compact

* Egypt’s Nile resources calculated as renewable share; actual availability constrained by treaty allocations.

Peru’s AR is 2.3× Egypt’s, 14× China’s, and 19× Spain’s. Unlike these nations, Peru has not implemented major inter-basin transfer infrastructure or comprehensive demand management programmes.

The Water Poverty Index (WPI) components reveal divergent challenges ( Figure 1B). The Pacific basin scores poorly on “Resources” (physical availability) but well on “Access” (infrastructure); the Amazon basin shows inverse patterns. This suggests that water poverty in Peru is driven by physical scarcity in the Pacific and economic scarcity (infrastructure deficits) in the Amazon.

### 3.3 Temporal trends (1990–2025)

Mann-Kendall tests reveal significant deteriorating trends in Pacific basin indicators ( Table 3):

**Table 3.** Temporal trend analysis (1990–2025).

Indicator	Basin	Kendall’s τ	p-value	Trend direction	Sen’s slope
WSI	Pacific	−0.78	<0.001	Decreasing	−18.4 m ^3^/capita/year
WSI	Amazon	−0.12	0.342	No trend	—
DP	Pacific	+0.84	<0.001	Increasing	+1.2% per year
DP	Amazon	+0.08	0.521	No trend	—

The Pacific basin WSI declined from 2,450 m ^3^/capita/year (1990) to 1,628 m ^3^/capita/year (2025), a 33.6% reduction. At current trends (Sen’s slope: −18.4/year), absolute scarcity (<1,000) will be reached by 2059 (95% CI: 2048–2071).

### 3.4 Sectoral allocation and efficiency

**Figure 2.**
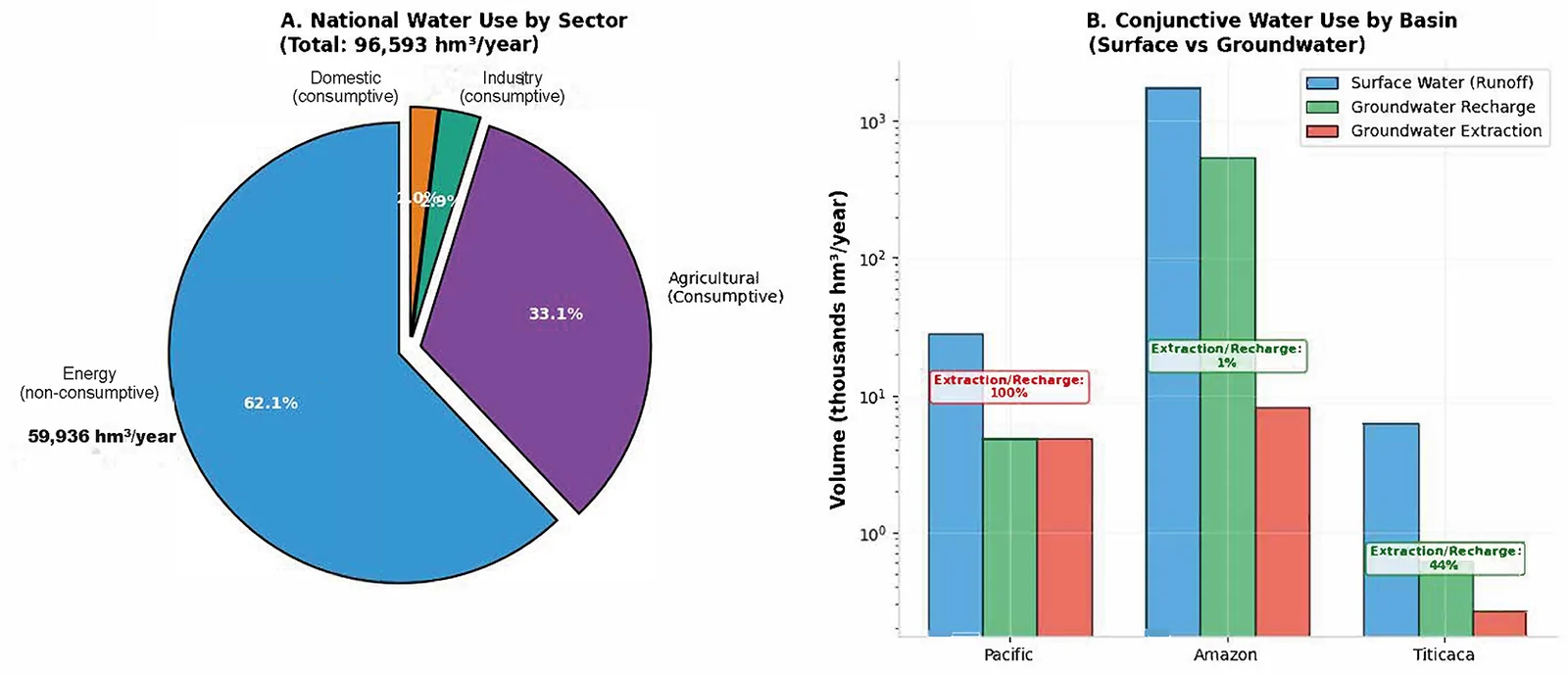
National water use distribution and conjunctive surface-groundwater analysis. (A) Sectoral water use distribution (total 96,593 hm ^3^/year). Energy use dominates (62.1%, non-consumptive), followed by agriculture (33.1%, consumptive), industry (2.9%), and domestic use (2.0%). (B) Conjunctive water use by basin showing surface water (runoff ), groundwater recharge, and groundwater extraction. Pacific basin extraction/recharge ratio: 100% (unsustainable). Amazon basin ratio: 1% (sustainable). Titicaca basin ratio: 44%.

National water consumption (96,593 hm ^3^/year) shows sectoral distribution ( Figure 2A):

• Energy (non-consumptive): 59,936 hm ^3^/year (62.1%)
• Agriculture (consumptive): 32,011 hm ^3^/year (33.1%)
• Industry (consumptive): 2,753 hm ^3^/year (2.9%)
• Domestic (consumptive): 1,887 hm ^3^/year (2.0%)

Agricultural water productivity (AWP) shows minimal inter-basin variation (Pacific: 76.0 ha/million m ^3^; Amazon: 78.5; Titicaca: 79.6), suggesting that efficiency differences do not drive pressure disparities. Instead, the absolute resource constraint in the Pacific basin (36,804 hm ^3^ vs. 1,893,055 hm ^3^ in Amazon) creates stress despite similar productivity.

Groundwater-surface water interactions reveal conjunctive use patterns ( Figure 2B). The Pacific basin extracts 4,844 hm ^3^/year groundwater recharge (100% of renewable recharge), indicating unsustainable mining of fossil aquifers in coastal areas. The Amazon basin utilises only 1.5% of rechargeable groundwater.

### 3.5 Economic compensation analysis

Economic compensation collections (S/205.5 million) show inequitable distribution ( Table 4):

**Table 4.** Economic compensation by Basin and Category (S/2024).

Basin	Agrarian	Non-Agrarian	Groundwater	Discharge	Total	% National	S/per hm ^3^ available
Pacific	17.97 M	66.31 M	24.30 M	18.23 M	126.81 M	61.7%	3.45
Amazon	1.39 M	44.71 M	7.93 M	22.42 M	76.45 M	37.2%	0.04
Titicaca	0.22 M	1.08 M	0.27 M	0.68 M	2.25 M	1.1%	0.35

The Pacific basin generates 86× more compensation per unit of water availability than the Amazon basin (S/3.45 vs. S/0.04 per hm ^3^). This inverse relationship between resource abundance and economic contribution indicates that compensation mechanisms tax scarcity rather than value ecosystem services.

Groundwater charges (S/32.5 million) are concentrated in the Pacific basin (74.8%), reflecting unsustainable extraction. Discharge fees (S/41.3 million) are split between Pacific (44.1%) and Amazon (54.2%), indicating pollution externalities in both basins.

### 3.6 Governance failure mechanisms

Political ecology analysis reveals three structural barriers:

*Accumulation by Dispossession:* Historical water rights allocation (1930s–1970s) concentrated 68% of Pacific basin surface water rights in 12 large agricultural export enterprises (sugar, rice, asparagus), whilst 450,000 smallholders share 23%. ^31^ This legacy constrains current reallocation options.

*Scalar Mismatch:* Watershed boundaries (159 hydrological units) intersect with 25 administrative regions and 1,874 municipal districts. The ANA’s basin-scale authority conflicts with regional government jurisdiction over infrastructure investment, creating coordination failures. ^32^

*Knowledge Exclusion:* Indigenous water management systems (comunidades campesinas, ayllus) maintain parallel governance structures in 34% of Andean watersheds. State IWRM planning excludes these systems, reducing implementation legitimacy. ^33^

## 4. Discussion

This study demonstrates that Peru’s water crisis is not characterised by national scarcity but by extreme hydrological asymmetry. While national renewable availability exceeds 56,000 m ^3^/capita/year, the Pacific basin remains below the Falkenmark water stress threshold (1,700 m ^3^/capita/year), with Monte Carlo simulations confirming structural stress even under optimistic scenarios. This confirms that aggregated national indicators obscure subnational vulnerability, a limitation previously identified in global water security assessments. ^1, 7, 19^

The asymmetry ratio calculated for Peru (AR = 112.5) substantially exceeds comparable international cases, including Egypt, China, Spain and the United States. Countries facing lower asymmetry ratios have implemented large-scale structural responses such as inter-basin transfers (China’s South–North Water Transfer), virtual water trade (Egypt), or binding allocation compacts (Colorado River Compact). ^10, 28, 31^ Peru, by contrast, has not adopted a systemic redistribution strategy. This absence is particularly notable given that hydroclimatic studies project significant runoff reductions in glacier-fed Pacific basins under climate change scenarios. ^23, 26^ Without structural intervention, current declining trends in the Pacific basin WSI suggest that absolute scarcity (<1,000 m ^3^/capita/year) could be reached within decades.

The Water Poverty Index (WPI) results further nuance this diagnosis. The Pacific basin scores poorly on the “Resources” component but relatively well on “Access”, reflecting infrastructure concentration. Conversely, the Amazon basin exhibits abundant physical resources but weaker infrastructure and service provision. This duality aligns with conceptual distinctions between physical and economic water scarcity. ^1, 3^ It also highlights that IWRM implementation in Peru has focused more strongly on allocation and regulation than on territorial equity and infrastructure balancing. ^7, 34^

Groundwater dynamics intensify these concerns. The Pacific basin currently exploits 100% of estimated renewable recharge, suggesting mining of fossil aquifers in coastal zones. Recent satellite gravimetry studies confirm long-term groundwater depletion trends in Peru’s arid basins. ^31^ This pattern is emblematic of what hydrosocial cycle scholarship describes as the socio-political production of scarcity: institutional arrangements and economic priorities shape patterns of extraction beyond hydrological limits. ^30^ In contrast, the Amazon basin utilises only a fraction of its renewable groundwater, reflecting infrastructural constraints rather than hydrological deficit.

Economic compensation mechanisms reveal a further structural paradox. The Pacific basin generates 86 times more compensation revenue per unit of available water than the Amazon basin. Current pricing instruments therefore internalise scarcity costs but fail to recognise ecosystem service provision. Payment for ecosystem services (PES) schemes implemented in Andean headwaters, such as those in the Santa River basin, illustrate how upstream conservation can be economically valued. ^33^ However, these initiatives remain geographically limited and financially marginal relative to national compensation flows. Aligning compensation schemes with ecosystem service valuation principles could reduce territorial inequities while strengthening adaptive capacity.

The governance barriers identified—accumulation by dispossession, scalar mismatch, and knowledge exclusion—are consistent with documented limitations in Peruvian IWRM implementation. ^7, 14, 35^ Historical water rights concentration constrains redistribution; overlapping administrative jurisdictions undermine basin-scale coordination; and indigenous governance systems remain insufficiently integrated into state planning frameworks. ^33^ These structural constraints help explain why Peru, despite formal adoption of IWRM principles since 2009, has not achieved hydrological equity.

Taken together, the findings suggest that Peru’s water challenge is a governance asymmetry layered upon a hydrological asymmetry. Addressing it requires moving beyond infrastructure-centric responses toward integrated demand management, groundwater regulation, ecosystem service valuation, and multi-scalar institutional reform. Without such recalibration, climatic variability and demographic concentration will further entrench coastal scarcity while leaving Amazonian abundance underutilised and undervalued.

## 5. Conclusions

This study provides the first uncertainty-quantified, internationally benchmarked assessment of hydrological asymmetry in Peru. Four principal conclusions emerge:

1. *Extreme subnational asymmetry:* Peru exhibits one of the highest recorded asymmetry ratios globally (AR = 112.5), with the Pacific basin structurally water-stressed despite national abundance.
2. *Divergent scarcity typologies:* The Pacific basin experiences physical scarcity, whereas the Amazon basin faces economic and infrastructural scarcity, consistent with global water poverty frameworks. ^1, 3, 28^
3. *Unsustainable groundwater exploitation:* Full utilisation of renewable recharge in the Pacific basin signals long-term depletion risks, corroborated by recent geodetic evidence. ^31^
4. *Misaligned economic instruments:* Current compensation mechanisms disproportionately tax scarcity without valuing ecosystem services, reinforcing territorial inequities.

Policy implications follow directly from these findings. First, basin-scale demand management and irrigation efficiency improvements are imperative in the Pacific basin. Second, groundwater monitoring and abstraction control must be strengthened to prevent irreversible depletion. Third, compensation mechanisms should incorporate ecosystem service valuation principles, redistributing revenues toward hydrologically strategic headwater regions. Finally, effective IWRM requires institutional integration across administrative scales and recognition of indigenous water governance systems.

Peru’s case illustrates a broader lesson for water-rich yet spatially uneven nations: aggregate abundance does not guarantee water security. Without institutional mechanisms that reconcile hydrology, demography, and political economy, asymmetry becomes a driver of chronic stress. Future research should prioritise high-resolution groundwater mapping, climate scenario modelling, and evaluation of compensation redistribution impacts to support evidence-based reform.

## Data Availability

All data supporting this study are publicly available from:
• National Water Authority (ANA): *ANA Institutional Repository –* https://repositorio.ana.gob.pe/handle/20.500.12543/5714 (dataset: “El Agua en números 2024”) • Repository name: *Hydrological Asymmetry and Water Stress in Peru: Integrated Assessment of Pacific, Amazon and Titicaca Basins (ANA Official Datasets 2017–2025).* https://doi.org/10.5281/zenodo.18759777 The project contains the following underlying data:
○
Table 1_Water_supply_population_by_sources.xlsx (Population distribution and water resources by basin: Pacific, Amazon, and Titicaca; INEI Census 2017 and 2025 projections) ○
Table 2_Hydrological_cycle_parameters.xlsx (Hydrological cycle parameters: precipitation, evapotranspiration, aquifer recharge, and runoff by basin) ○
Table 3_Sectoral_water_use_pressure.xlsx (Sectoral water use: agricultural, industrial, population, and energy; degree of pressure on water resources) ○
Table 4_Economic_compensation_water.xlsx (Economic compensation for water use by basin and category: agrarian, non-agrarian, groundwater, and discharge) Data are available under the terms of the Creative Commons Zero “No rights reserved” data waiver (CC0 1.0 Public domain dedication). ^36^
