# Supplementary material for: Hydrological asymmetry and water stress in Peru: An integrated assessment of resource distribution, anthropogenic pressure, and governance gaps across three drainage basins
Source: F1000Res. 2026 Mar 30;15:456. [Version 1] doi: 10.12688/f1000research.176208.1 (PMC13560054; doi:10.12688/f1000research.176208.1)
Supplement: Peer review history

# Reviewer report: Reviewer response for version 1

**DOI:** 10.5256/f1000research.194245.r503313

**Published:** 2026-08-08

**Authors:** Elmer Calizaya

**Affiliations:** 1 Universidad Nacional del Altiplano, Puno, Puno, Peru

I believe this manuscript presents an important and relevant analysis of the evolution of the Water Stress Index (WSI), particularly because it integrates trend analysis with the evolution of uncertainty through Monte Carlo simulations. The observations made are primarily methodological and related to presentation and can be addressed without substantially altering the study’s approach. Among the main revisions are clarifying and establishing a clear correspondence between the research questions and results, justifying the Monte Carlo distributions used, revising the trend of the Pacific WSI in Table 3, clarifying the meaning of “86x more” and improving the resolution, font size, and captions in Figures 1 and 2. I believe that addressing these comments will enhance the clarity, consistency, and scientific rigor of the manuscript.

Does the paper provide a comprehensive overview of the policy and the context of its implementation in a way which is accessible to a general reader?

Yes

Is the discussion on the implications clearly and accurately presented and does it cite the current literature?

Yes

Are the recommendations made clear, balanced, and justified on the basis of the presented arguments?

Partly

Reviewer Expertise:

Hydrology, Water Resources, Geomatics, Geographic Information System, Climate Change.

I confirm that I have read this submission and believe that I have an appropriate level of expertise to confirm that it is of an acceptable scientific standard.

# Reviewer report: Reviewer response for version 1

**DOI:** 10.5256/f1000research.194245.r503320

**Published:** 2026-07-30

**Authors:** Edgar Virgilio Bedoya Justo

**Affiliations:** 1 Universidad Nacional de Moquegua, Perane, Moquegua, Peru

The manuscript provides a comprehensive and accessible overview of Peru's hydrological asymmetry, effectively integrating hydrological, demographic, and governance dimensions into a coherent analytical framework. The authors successfully demonstrate that the country's water-related challenges arise primarily from the spatial mismatch between water availability and population distribution rather than from absolute national water scarcity. The combination of quantitative statistical analyses, uncertainty assessment, and institutional evaluation offers a balanced and policy-relevant contribution to the understanding of water security in Peru.

 The policy background and implementation context are clearly presented and are sufficiently detailed for both specialists and a broader scientific audience. The discussion of the three drainage basins is well structured and supported by appropriate quantitative evidence, allowing readers to understand the complex interactions between hydrological conditions, socio-economic pressures, and governance arrangements.

 The analysis of the implications is clearly articulated and supported by relevant and up-to-date scientific literature. The application of non-parametric trend analysis and uncertainty propagation strengthens the credibility of the findings, while the integration of the political ecology perspective provides valuable context for interpreting the observed hydrological disparities. References are appropriate, current, and adequately support the arguments presented throughout the manuscript.

 The recommendations are clear, balanced, and logically derived from the evidence presented. They appropriately emphasize the need for more integrated basin-scale water governance, improved institutional coordination, and policies that address regional inequalities in water availability and management. These recommendations are realistic, policy-oriented, and consistent with the results obtained in the study.

 Overall, this is a scientifically sound and well-organized manuscript that makes a meaningful contribution to the literature on integrated water resources management, hydrological sustainability, and environmental governance. The methodology is appropriate for the stated objectives, the interpretation of the results is well supported by the data, and the conclusions are justified by the analyses presented. Although the manuscript is scientifically robust, a few minor editorial refinements would further improve its readability. The discussion could benefit from smoother transitions between the quantitative findings and their policy implications, while brief clarifications accompanying the statistical methods would make the manuscript more accessible to readers from different disciplinary backgrounds. In addition, careful editing to eliminate minor redundancies and ensure consistent terminology, abbreviations, and formatting throughout the text would enhance clarity and presentation. These suggestions are purely editorial and do not affect the scientific soundness, methodological rigor, or validity of the conclusions.

 In my opinion, the manuscript is suitable for indexing after routine editorial revision, as it represents a valuable contribution to both the scientific community and policy makers interested in sustainable water governance and climate resilience.

Does the paper provide a comprehensive overview of the policy and the context of its implementation in a way which is accessible to a general reader?

Yes

Is the discussion on the implications clearly and accurately presented and does it cite the current literature?

Yes

Are the recommendations made clear, balanced, and justified on the basis of the presented arguments?

Yes

Reviewer Expertise:

Environment and sustainable development

I confirm that I have read this submission and believe that I have an appropriate level of expertise to confirm that it is of an acceptable scientific standard.
